# Molecular Characterization and Phylogenetic Analysis of New Variants of the Porcine Epidemic Diarrhea Virus in Gansu, China in 2012

**DOI:** 10.3390/v5081991

**Published:** 2013-08-15

**Authors:** Yufei Tian, Zhijun Yu, Kaihui Cheng, Yuxiu Liu, Jing Huang, Yue Xin, Yuanguo Li, Shengtao Fan, Tiecheng Wang, Geng Huang, Na Feng, Zhenguo Yang, Songtao Yang, Yuwei Gao, Xianzhu Xia

**Affiliations:** 1 College of Animal Science and Veterinary Medicine, Jilin University, Changchun 130062, China; 2 Key Laboratory of Jilin Province for Zoonosis Prevention and Control, Military Veterinary Research Institute of Academy of Military Medical Sciences, Changchun, 130122, China; 3 Institute of Laboratory Animal Sciences, Chinese Academy of Medical Sciences and Peking Union Medical College, Beijing 100021, China; 4 Dairy Cattle Research Center, Shandong Academy of Agricultural Sciences, Jinan 250132, China

**Keywords:** molecular characterization, spike glycoprotein gene, phylogenetic analysis, porcine epidemic diarrhea virus (PEDV)

## Abstract

Between January 2012 and March 2012, the infection rates of porcine epidemic diarrhea virus (PEDV) increased substantially in vaccinated swine herds in many porcine farms in Gansu Province, China. The spike (S) glycoprotein is an important determinant for PEDV biological properties. To determine the distribution profile of PEDV outbreak strains, we sequenced the full-length S gene of five samples from two farms where animals exhibited severe diarrhea and high mortality rates. Five new PEDV variants were identified, and the molecular diversity, phylogenetic relationships, and antigenicity analysis of Gansu field samples with other PEDV reference strains were investigated. A series of insertions, deletions, and mutations in the S gene was found in five PEDV variants compared with classical and vaccine strains. These mutations may provide stronger pathogenicity and antigenicity to the new PEDV variants that influenced the effectiveness of the CV777-based vaccine. Our results suggest that these new PEDV variant strains in Gansu Province might be from South Korean or South China, and the effectiveness of the CV777-based vaccine needs to be evaluated.

## 1. Introduction

Porcine epidemic diarrhea virus (PEDV), a member of *Coronaviridae*, is an enveloped, single-stranded RNA genome with a 5' cap and a 3' polyadenylated tail. The size of its genome is approximately 28 Kb [1]. The genome comprises a 5' untranslated region (UTR); a 3' UTR; at least seven open reading frames (ORFs) that encode four structural proteins, namely, spike (S), envelope (E), membrane (M), and nucleocapsid (N); and three non-structural proteins, namely, replicases 1a and 1ab as well as ORF3 [2]. The PEDV S protein is a type I glycoprotein composed of 1,383 amino acids (aa). Similar to other coronavirus S proteins, the PEDV S protein is a glycoprotein peplomer (surface antigen) on the viral surface and contains four neutralizing epitopes (499–638, 748–755, 764–771, and 1,368–1,374 aa) [3,4,5]. The PEDV S protein has a pivotal function in regulating interactions with specific host cell receptor glycoproteins to mediate viral entry [6]. Thus, the S glycoprotein is a primary target for the development of vaccines against PEDV. The S glycoprotein is also the major envelope glycoprotein of the virion, which serves as an important viral component to understand the genetic relationships of different PEDV strains and the epidemiological status of PEDV in the field [2,7,8,9].

Porcine epidemic diarrhea (PED), caused by PEDV, is an acute, highly contagious, and devastating swine disease that is characterized by acute enteritis and lethal watery diarrhea, followed by dehydration frequently leading to high mortality in piglets [10,11]. PED was first observed among English fattening pigs in 1971 [10] but has increasingly become a problem in several Asian countries, including Korea [12], China [8,9,13], Japan [14], and Thailand [15]. In China, PEDV was first isolated in 1973 [9]. Almost two decades later, its prevalence has become a problem of the swine industry in China, but until 2010, the prevalence of PEDV infection was relatively low with only sporadic outbreaks. However, in late 2010, a remarkable increase in PED outbreaks occurred in the pig-producing provinces [9,16]. PED that occurred in several porcine farms and caused severe economic loss between January 2012 and March 2012 in Gansu Province, China was investigated in this study. The affected pigs exhibited watery diarrhea, dehydration, and thin-walled intestines. The disease progressed to death within four days. Pigs of all ages were affected and exhibited diarrhea and loss of appetite with different degrees of severity, which were determined to be age dependent. Among the suckling piglets, 100% became ill. Pigs >7 days of age experienced mild diarrhea and anorexia, which completely resolved within a few days. To identify the PEDV strain(s) responsible for the recent outbreak in Gansu, where located in west China, the east by Shanxi province, the south of Sichuan province, the west of Xinjiang province, and the north of Inner Mongolia province, we sequenced the full-length S gene of the isolates obtained from the diarrhea samples collected from pigs in two affected pig farms. One farm named Yongjing Tai Chi Breed Co., Ltd (Yongjing, China), and another named Hoggery of Science and Technology Breed Park of Jiugang Hongfeng Company (Jiayuguan, China).

## 2. Results and Discussion

### 2.1. Sequence Analysis of the S Gene

**Figure 1:**
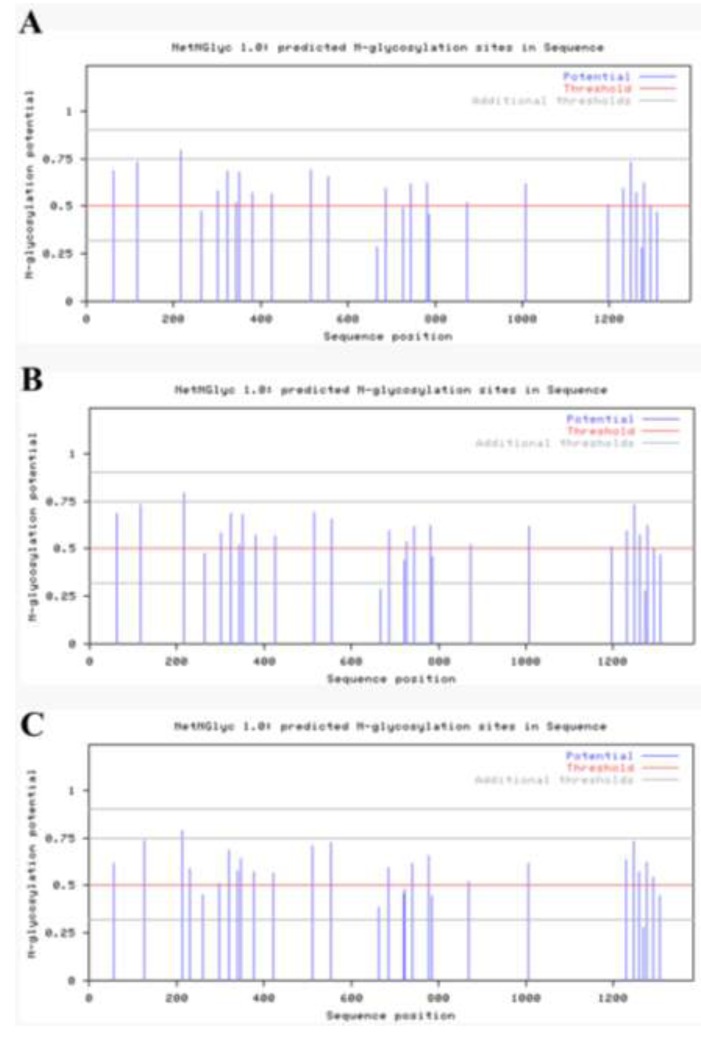
(**A**) S proteins of the JY5C, JY6C, JY7C and JY3F strains contained the same Asn-Xaa-Ser/Thr sequons and asparagines predicted to be *N*-glycosylated; (**B**) *N*-glycosylated prediction of the S protein of YJ7C strain; (**C**) *N*-glycosylated prediction of the S protein of CV777 strain.

**Figure 2:**
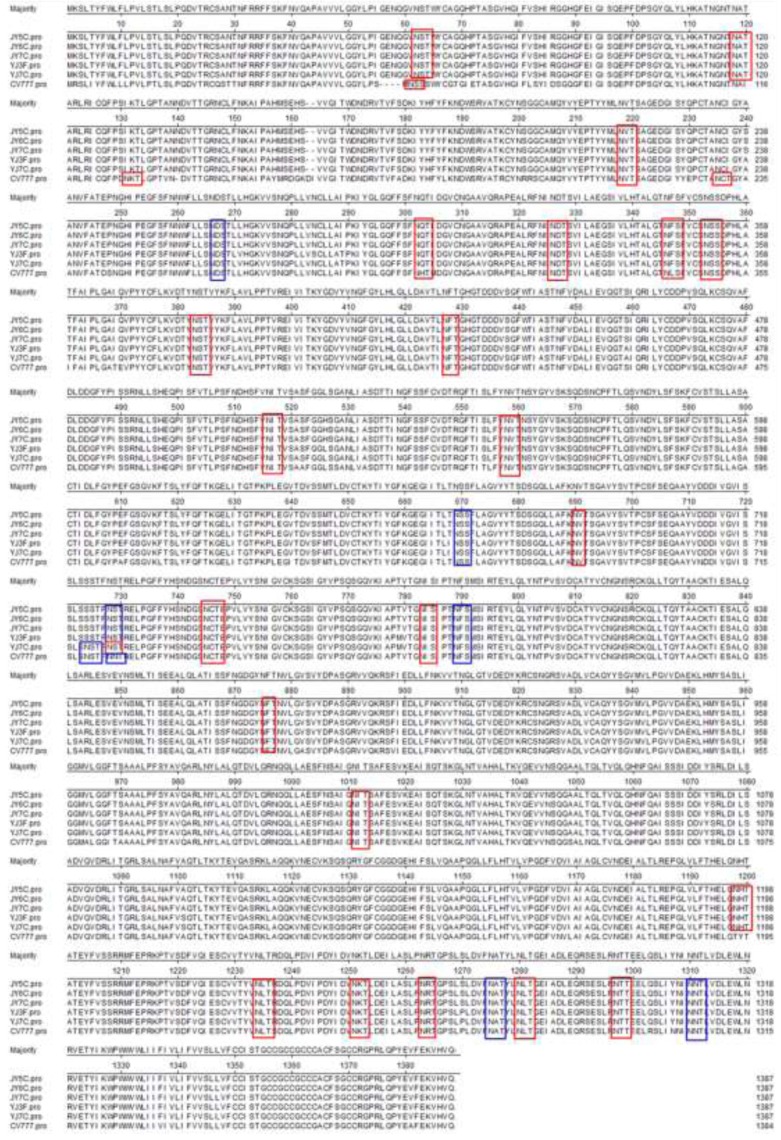
Amino acid alignment of Asn-Xaa-Ser/Thr sequons and asparagines predicted to be *N*-glycosylated of the JY5C, JY6C, JY7C, YJ3F and YJ7C strains’ S protein. Both blue boxes and red boxes stand for the Asn-Xaa-Ser/Thr sequons, but only red boxes stand for asparagines predicted to be *N*-glycosylated.

The nucleotide sequences of the S region were 4,161 bp long for JY5C, JY6C, JY7C, YJ3F, and YJ7C. Their S proteins were 1,386 aa long with a predicted Mr of 151.7 KDa. The S protein of JY5C, JY6C, JY7C, and YJ3F contained 28 Asn-Xaa-Ser/Thr sequons and 22 asparagines predicted to be *N*-glycosylated (Figure 1A,B). The S protein of the YJ7C strain contained 29 Asn-Xaa-Ser/Thr sequons and 23 asparagines predicted to be *N*-glycosylated (Figure 1C). However, the S protein of the CV777 vaccine strain contained 29 Asn-Xaa-Ser/Thr sequons and 22 asparagines predicted to be *N*-glycosylated (Figure 1D). For JY5C, JY6C, JY7C, and YJ3F, four (from N to V at 57, from N to I at 127, from T to I at 232, and from N to S at 719) of the changes in the predicted amino acid sequence destroyed *N*-linked glycosylation sites, whereas another three changes (from S to N at 58, from I to T at 116, and from T to N at 1193) created three new glycosylation sites compared with the vaccine strain CV777 (Figure 2). For the YJ7C strain, three amino acid changes (from N to V at 57, from N to I at 127, and from T to I at 232) destroyed *N*-linked glycosylation sites and another three changes (from S to N at 58, from I to T at 116, and from T to N at 1193) created three new glycosylation sites compared with the vaccine strain CV777 (Figure 2). The changes in the *N*-linked glycosylation sites between the Gansu PEDV strains from our study and the vaccine strain may influence their pathogenicity and antigenicity and should be researched in the future. 

### 2.2. Nucleotide and Deduced Amino Acid Sequence Homology

The nucleotide and deduced amino acid sequence homology results are described in Table 1. The nucleotide sequence of the five strains from our study (JY5C, JY6C, JY7C, YJ3F, and YJ7C) had 99.3% to 100% nucleotide sequence identity to one another, and their deduced amino acids had 99.0% to 100% identity to one another. The S genes’ nucleotide and deduced amino acid identities of five strains from our study (JY5C, JY6C, JY7C, YJ3F, and YJ7C) with the other PEDV reference strains are described in Table 2. The PEDV strain that had the highest DNA sequence identity to our PEDV strains was CH8 (one Chinese PEDV strain), which had 98.4% identity and the deduced amino acids had more than 98.0% identity. However, the nucleotide sequence of our PEDV strains had only 93.8% to 93.9% identity to the CV777 vaccine strain, and their deduced amino acids had 93.6% to 93.7% identity to the CV777 vaccine strain. The nucleotide sequence of our PEDV strains had lower identity (93.3% to 95.7%) to the previous domestic strains (DX, LZC, LJB-03, JS-2004-2, and CHS) and their deduced amino acids had 92.6% to 95.7% identity to the previous domestic strains (DX, LZC, LJB-03, JS-2004-2, and CHS). The nucleotide sequence of our PEDV strains also had low identity (93.2% to 94.6% and 93.7% to 93.8%) to the Japanese strains (MK, NK, and KH) and the European strain (Br1-87), respectively, and their deduced amino acids had 93.1% to 94.2% and 93.4% to 93.6% identities to the Japanese strains (MK, NK, and KH) and the Europe strain (Br1-87), respectively. The nucleotide sequence of our PEDV strains had higher identity (94.7% to 97.1%) to seven South Korean strains (KNU-0801, KNU-0802, KNU-0901, KNU-0902, KNU-0903, KNU-0904, and KNU-0905), and their deduced amino acids had 94.1% to 96.8% identity. 

**Table 1:** Comparison of the nucleotide and deduced amino acid sequences of S genes of PEDV (porcine epidemic diarrhea virus) reference strains and PEDV outbreak in Gansu, China.

Virus strian	1	2	3	4	5	6	7	8	9	10	11	12	13	14	15	16	17	18	19	20	21	22	23	24	25	26	27	28	29	30	31	32	33	34	35	36	37
1 JY5C		100.0	100.0	99.3	99.3	97.6	95.1	93.4	93.4	95.1	95.2	94.4	98.4	97.7	94.6	95.3	97.5	95.0	93.3	94.9	95.7	93.4	95.1	97.1	95.9	95.0	95.8	94.7	96.5	93.8	93.2	93.6	93.2	94.5	94.3	93.8	93.7
2 JY6C	100.0		100.0	99.3	99.3	97.6	95.1	93.4	93.4	95.1	95.2	94.4	98.4	97.7	94.6	95.3	97.5	95.0	93.3	94.9	95.7	93.4	95.1	97.1	95.9	95.0	95.8	94.7	96.5	93.8	93.2	93.6	93.2	94.5	94.3	93.8	93.7
3 JY7C	100.0	100.0		99.3	99.3	97.6	95.1	93.4	93.4	95.1	95.2	94.4	98.4	97.7	94.6	95.3	97.5	95.0	93.3	94.9	95.7	93.4	95.1	97.1	95.9	95.0	95.8	94.7	96.5	93.8	93.2	93.6	93.2	94.5	94.3	93.8	93.7
4 YJ3F	99.0	99.0	99.0		100.0	97.6	95.1	93.5	93.5	95.1	95.2	94.4	98.4	97.7	94.7	95.3	97.5	95.0	93.3	94.9	95.6	93.5	95.2	97.0	95.8	94.9	95.7	94.7	96.7	93.8	93.3	93.7	93.2	94.6	94.2	93.8	93.8
5 YJ7C	99.0	99.0	99.0	99.9		97.6	95.1	93.6	93.5	95.1	95.2	94.4	98.4	97.7	94.7	95.4	97.6	95.0	93.4	94.9	95.6	93.6	95.2	97.0	95.9	95.0	95.8	94.8	96.7	93.9	93.4	93.7	93.2	94.6	94.3	93.9	93.8
6 CH1	97.9	97.9	97.9	97.6	97.6		94.5	93.4	93.3	94.5	94.6	95.8	98.4	98.5	94.2	94.9	97.1	94.6	92.9	94.5	94.9	93.0	94.8	96.5	95.4	94.8	95.3	94.5	96.2	93.5	93.2	93.3	93.1	94.3	94.1	93.4	93.4
7 CH2	95.1	95.1	95.1	94.9	94.9	94.6		95.8	95.8	99.5	99.6	97.6	95.3	94.0	96.8	95.9	94.4	97.3	95.2	97.2	98.4	95.6	93.1	93.9	93.8	92.9	93.8	92.8	93.6	95.8	95.6	92.6	95.2	93.0	92.7	95.7	95.6
8 CH3	92.8	92.8	92.8	92.8	93.0	92.9	95.3		99.7	95.8	96.0	95.8	93.7	93.1	97.6	96.6	94.4	96.0	96.2	95.9	96.3	96.6	94.0	93.4	93.5	93.0	93.4	94.2	93.6	96.8	99.5	93.8	97.6	94.2	94.2	96.8	96.7
9 CH4	92.8	92.8	92.8	92.8	93.0	92.9	95.3	99.4		95.8	95.9	95.7	93.6	93.0	97.5	96.6	94.3	95.9	96.2	95.8	96.2	96.6	94.0	93.3	93.4	92.9	93.3	94.1	93.5	96.8	99.4	93.7	97.5	94.2	94.1	96.8	96.7
10 CH5	94.6	94.6	94.6	94.5	94.5	94.1	99.1	94.9	94.9		99.6	97.6	95.3	94.0	96.7	95.9	94.3	97.3	95.2	97.2	98.4	95.6	93.1	93.9	93.8	92.9	93.8	92.8	93.6	95.8	95.6	92.6	95.2	93.0	92.7	95.7	95.6
11 CH6	95.1	95.1	95.1	94.9	94.9	94.6	99.6	95.4	95.4	99.1		97.7	95.4	94.1	96.9	96.1	94.5	97.4	95.4	97.3	98.5	95.7	93.3	94.0	93.9	93.0	93.9	93.0	93.8	96.0	95.7	92.8	95.3	93.2	92.9	95.9	95.8
12 CH7	94.6	94.6	94.6	94.3	94.3	95.6	97.5	95.3	95.3	97.0	97.5		94.7	95.1	96.5	95.6	94.0	97.2	94.9	97.0	97.8	95.3	92.8	93.5	93.5	92.9	93.5	92.6	93.4	95.4	95.5	92.3	95.1	92.7	92.4	95.4	95.3
13 CH8	98.1	98.1	98.1	98.0	98.0	98.6	95.0	92.9	92.9	94.6	95.0	94.6		97.5	94.6	95.3	97.4	94.9	93.3	94.8	95.6	93.4	95.2	96.9	95.8	95.0	95.7	94.8	96.5	93.8	93.5	93.5	93.4	94.7	94.5	93.8	93.7
14 CHGD01	98.1	98.1	98.1	97.9	97.9	98.2	93.9	92.7	92.7	93.5	93.9	94.6	97.4		94.3	95.0	97.1	94.4	92.9	94.4	94.7	92.9	94.6	96.5	95.3	94.5	95.3	94.4	96.3	93.4	92.9	93.4	92.9	94.2	93.9	93.4	93.4
15 CH-FJND-1-2011	93.8	93.8	93.8	94.0	94.1	93.6	96.0	97.3	97.2	95.6	96.1	95.7	93.8	93.7		98.9	96.7	96.8	95.9	96.7	97.3	96.0	93.2	94.2	94.2	93.2	94.1	93.3	94.1	96.4	97.3	93.1	96.1	93.4	93.3	96.4	96.3
16 CH-FJND-2-2011	95.1	95.1	95.1	95.2	95.4	94.9	95.0	96.1	96.0	94.6	95.1	94.7	94.8	94.9	98.6		97.5	96.1	95.2	96.0	96.5	95.3	93.6	94.9	94.5	93.6	94.5	93.7	94.7	95.7	96.4	93.4	95.3	93.8	93.5	95.8	95.7
17 CH-FJND-3-2011	97.0	97.0	97.0	97.1	97.3	96.8	93.8	93.8	93.8	93.3	93.8	93.4	96.7	96.6	96.3	97.5		94.5	93.6	94.5	94.9	93.7	95.1	96.9	95.9	95.0	95.8	95.1	96.5	94.1	94.2	93.9	93.7	94.9	94.8	94.1	94.1
18 DX	95.3	95.3	95.3	95.2	95.2	94.8	97.2	95.4	95.4	96.9	97.2	97.0	95.0	94.6	96.5	95.5	94.4		95.5	99.1	98.1	95.9	93.2	94.2	94.3	93.4	94.2	93.1	94.0	96.0	95.7	93.0	95.3	93.2	93.0	96.0	95.9
19 LZC	92.8	92.8	92.8	92.6	92.8	92.3	94.8	94.9	94.9	94.4	95.0	94.6	92.3	92.6	94.9	94.2	92.7	94.8		95.4	95.9	95.8	93.4	93.1	93.2	93.0	93.2	93.5	93.5	99.4	96.0	93.8	95.5	93.7	93.3	99.5	99.4
20 LJB-03	94.9	94.9	94.9	94.8	94.8	94.4	96.9	95.2	95.2	96.6	96.9	96.7	94.6	94.3	96.0	95.0	93.8	98.6	94.5		98.0	95.8	93.2	94.2	94.1	93.3	94.1	93.1	93.9	96.0	95.6	92.9	95.2	93.1	92.9	96.0	95.9
21 JS-2004-2	95.7	95.7	95.7	95.5	95.5	94.9	98.0	95.8	95.8	97.6	98.0	97.6	95.3	94.7	96.7	95.7	94.4	98.0	95.4	97.8		96.2	93.6	94.7	94.6	93.7	94.6	93.3	94.4	96.4	96.1	93.1	95.7	93.4	93.2	96.4	96.3
22 CHS	93.3	93.3	93.3	93.3	93.5	93.0	95.4	96.1	96.1	95.2	95.5	95.2	93.1	93.0	95.6	94.6	93.3	95.7	95.2	95.4	96.1		94.0	93.9	93.9	93.4	93.8	93.7	93.7	96.3	96.3	93.5	95.8	94.0	93.4	96.4	96.3
23 KUN-0901	94.7	94.7	94.7	94.7	94.8	94.4	92.9	93.4	93.3	92.5	93.1	92.5	94.7	94.4	92.7	93.5	94.5	93.0	92.8	92.7	93.3	93.6		95.4	95.5	95.7	95.4	97.9	95.8	93.9	93.7	96.0	93.4	96.7	94.7	93.9	93.9
24 KUN-0902	96.4	96.4	96.4	96.1	96.3	95.9	93.1	92.4	92.4	92.9	93.1	92.8	96.0	95.9	93.0	94.2	96.0	93.6	92.3	93.2	94.1	93.5	94.5		97.8	95.3	97.6	94.9	96.5	93.6	93.1	93.8	92.9	94.6	94.3	93.6	93.6
25 KUN-0903	95.0	95.0	95.0	94.8	95.0	94.4	93.1	92.6	92.5	92.8	93.3	93.1	94.7	94.4	93.1	94.1	94.8	93.6	92.3	93.1	94.1	93.5	95.1	96.5		96.8	99.8	95.1	95.8	93.7	93.2	94.1	92.9	95.1	94.6	93.8	93.7
26 KUN-0904	94.1	94.1	94.1	94.0	94.2	93.9	92.5	92.0	92.0	92.1	92.8	92.4	93.9	93.8	92.3	93.3	93.9	92.8	91.9	92.5	93.1	92.8	95.3	94.0	96.5		96.7	95.1	96.2	93.5	92.7	93.9	92.3	95.0	94.2	93.5	93.5
27 KUN-0905	94.7	94.7	94.7	94.6	94.7	94.2	93.1	92.4	92.3	92.8	93.2	93.0	94.4	94.2	92.9	93.8	94.6	93.4	92.2	92.9	94.0	93.3	95.0	96.2	99.6	96.4		95.0	95.7	93.6	93.1	94.0	92.9	94.9	94.5	93.7	93.6
28 KUN-0801	94.1	94.1	94.1	94.1	94.2	94.2	92.3	93.6	93.5	91.9	92.5	92.2	94.2	94.2	92.8	93.6	94.5	92.8	92.7	92.4	93.0	93.3	97.9	94.1	94.7	94.5	94.4		95.4	94.1	93.9	97.8	93.6	97.9	95.0	94.1	94.0
29 KUN-0802	96.6	96.6	96.6	96.6	96.8	96.2	93.5	92.8	92.8	93.1	93.6	93.2	96.3	96.5	93.6	94.7	96.2	93.8	92.7	93.4	94.2	93.4	95.4	96.4	95.5	94.9	95.2	95.5		94.0	93.3	94.2	93.0	95.1	94.6	94.1	94.0
30 Parent DR13	93.7	93.7	93.7	93.6	93.7	93.3	95.7	95.9	95.9	95.4	96.0	95.4	93.3	93.5	95.7	95.0	93.5	95.7	98.9	95.4	96.2	96.0	93.6	93.1	93.1	92.7	93.0	93.6	93.5		96.6	94.3	96.0	94.2	93.9	99.9	99.8
31 Attenuated DR13	92.8	92.8	92.8	92.8	93.0	92.8	95.2	99.2	99.1	94.8	95.3	95.2	92.8	92.6	97.0	95.9	93.7	95.3	94.8	95.0	95.7	95.8	93.1	92.3	92.5	92.0	92.3	93.3	92.8	95.7		93.5	97.3	94.0	93.9	96.5	96.4
32 Chinju99	92.5	92.5	92.5	92.5	92.6	92.3	90.9	92.0	92.0	90.5	91.2	90.9	92.3	92.5	91.6	92.2	92.8	91.5	92.0	91.2	91.8	92.0	95.4	92.5	92.8	92.7	92.6	97.3	93.6	92.8	91.9		93.3	96.3	93.8	94.4	94.4
33 MK	93.4	93.4	93.4	93.1	93.3	93.5	94.9	96.6	96.5	94.5	94.9	95.1	93.3	93.3	95.4	94.7	93.5	95.2	94.8	94.9	95.5	95.5	93.3	93.1	92.9	92.2	92.7	93.8	93.4	95.6	96.3	92.5		93.8	93.7	96.1	96.0
34 NK	93.9	93.9	93.9	94.1	94.2	93.9	92.8	93.7	93.6	92.3	93.0	92.6	94.0	93.8	93.0	93.7	94.2	93.1	92.9	92.7	93.5	93.8	96.3	93.7	94.4	94.2	94.2	97.5	94.7	93.8	93.5	95.5	94.1		95.2	94.2	94.2
35 KH	93.9	93.9	93.9	93.7	93.9	93.8	92.7	93.1	93.0	92.3	92.8	92.3	94.0	93.7	92.3	93.2	93.8	92.7	92.3	92.3	92.9	93.1	94.2	93.4	94.1	93.4	93.9	94.6	94.1	93.3	92.9	92.8	93.7	94.9		93.8	93.8
36 CV777	93.7	93.7	93.7	93.6	93.7	93.3	95.7	95.9	95.9	95.3	95.9	95.4	93.3	93.6	95.9	95.2	93.6	95.7	99.1	95.4	96.2	96.2	93.7	93.2	93.2	92.8	93.1	93.6	93.6	99.7	95.7	93.0	95.7	93.8	93.2		99.9
37 Br1-87	93.6	93.6	93.6	93.4	93.6	93.1	95.4	95.6	95.6	95.1	95.6	95.2	93.1	93.4	95.6	94.9	93.5	95.4	98.8	95.2	96.0	95.9	93.6	93.1	93.1	92.7	93.0	93.5	93.5	99.4	95.4	93.0	95.4	93.8	93.1	99.7	

Nucleotide identity (%) in upper triangle; Deduced amino acid identity (%) in lower triangle.

**Table 2:** The S genes’ nucleotide and deduced amino acid identities of five strains from our study (JY5C, JY6C, JY7C, YJ3F, and YJ7C) with the other PEDV reference strains.

The other strains	Nucleotide identities	Deduced amino acid identities
CH8 (one Chinese PEDV strain)	98.40%	98.0%-98.1%
CV777 vaccine strain	93.8%–93.9%	93.6%–93.7%
Previous domestic strains (DX, LZC, LJB-03, JS-2004-2, and CHS)	93.3%–95.7%	92.6%–95.7%
Japanese strains (MK, NK, and KH)	93.2%–94.6%	93.1%–94.2%
European strain (Br1-87)	93.7%–93.8%	93.4%–93.6%
South Korean strains (KNU-0801, KNU-0802, KNU-0901, KNU-0902, KNU-0903, KNU-0904, and KNU-0905)	94.7%–97.1%	94.1%–96.8%

### 2.3. Phylogenetic Analysis of the S Gene

**Figure 3:**
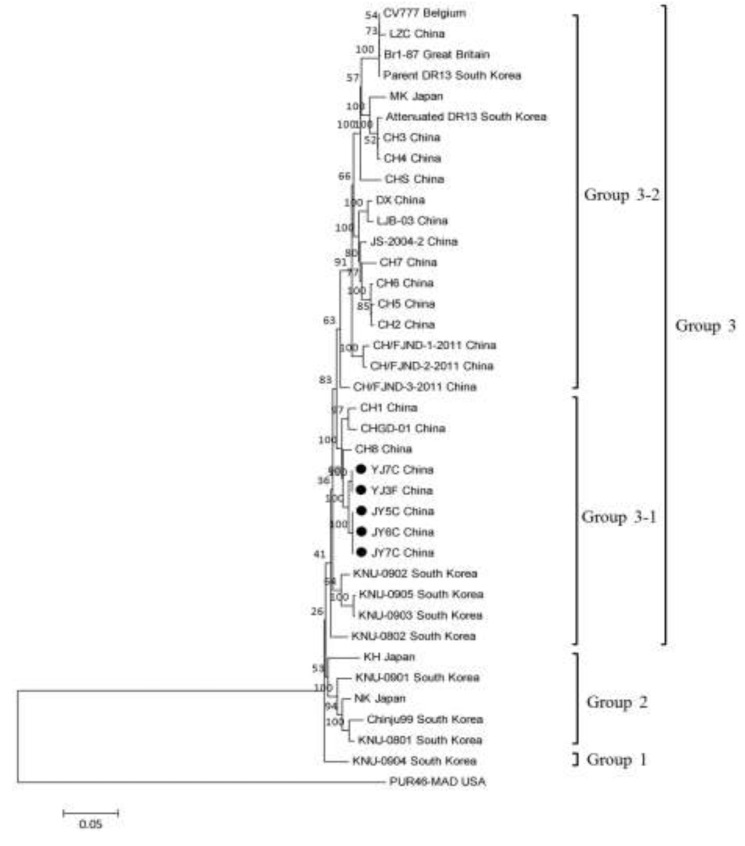
Phylogenetic trees of PEDV strains generated by the neighbor-joining method with nucleotide sequences of the full-length spike genes. Bootstrapping with 1,000 replicates was performed to determine the percentage reliability for each internal node. PUR46-MAD is an out group control. Horizontal branch lengths are proportional to genetic distances between PEDV strains. Black circles indicate PEDV isolates from the 2012 outbreak in Gansu Province, China. Scale bar indicates nucleotide substitutions per site.

**Figure 4:**
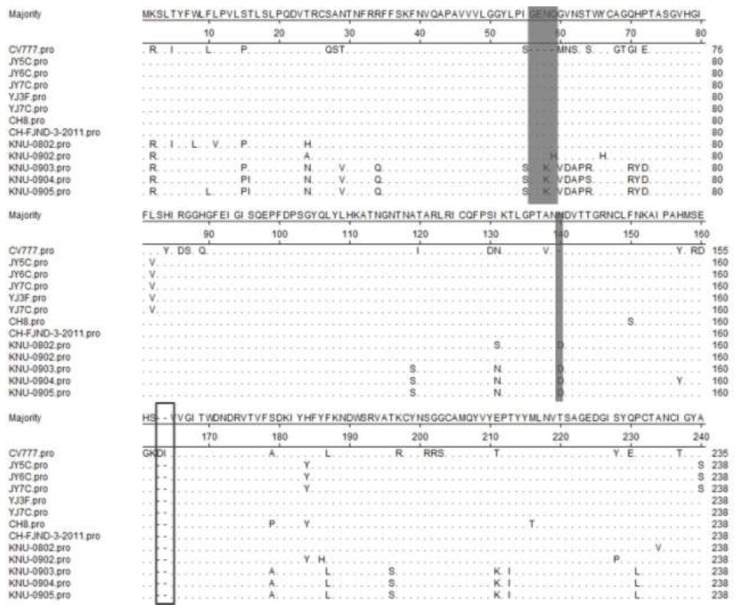
Alignment of amino terminal 1–238 amino acid of S proteins of Gansu PEDV strains and reference strains. Ellipses represent the consensus amino acids. Boxes indicate deleted amino acids compared with CV777. Shadows indicate the inserted amino acids compared with CV777.

Phylogenetic analysis of the nucleotide sequences of the S gene revealed three major clusters, and the third group has two subgroups (3-1 and 3-2). All PEDVs isolated from China in our study belonged to subgroup 3-1 (Figure 3). Group 1 comprised one strain from South Korea (KNU-0904). Group 2 comprised three strains from South Korea (KNU-0801, KNU-0901, and Chinju 99) and two strains from Japan (NK and KH). Subgroup 3-1 comprised five strains from our study (JY5C, JY6C, JY7C, YJ3F, and YJ7C), three strains (CH1, CH8, CHGD-01) that were identified from China in 2011 and four strains from South Korea KNU-0802, KNU-0902, KNU-0903, and KNU-0905). Group 3-2 comprised 15 strains from China (vaccine strain CV777, CH2, CH3, CH4, CH5, CH6, CH7, CHS, LZC, DX, LJB-03, JS-2004-2, CH/FJND-1-2011, CH/FJND-2-2011, and CH/FJND-3-2011), two strains from South Korea (parent DR13 and attenuated DR13), one strain from Great Britain (Br1-87), and one strain from Japan (MK). The five variant strains from our study (JY5C, JY6C, JY7C, YJ3F, and YJ7C), two strains (CH8 and CH/FJND-3-2011) that were identified from China, and five PEDV isolates from South Korea (KNU-0802, KNU-0902, KNU-0903, and KNU-0905) shared a 4-aa insertion (at positions 56–59 of the S protein), 1-aa insertion (at position 140 of the S protein), and 2-aa deletions (at positions 163–164 of the S protein), compared with CV777 (Figure 4). Our results indicate that the North Chinese PEDV strains from our study had a close relationship with the South Korean strains and mapped phylogenetically to the same branch. However, they differed genetically from the European strains (including the vaccine strain CV777) and the early domestic strains. Similar to the result by Li *et al.* [9], the appearance of strains from China similar to those from South Korea and their function in the recent PED outbreak in South China, should be further investigated.

### 2.4. Antigenicity Analysis of the S Gene

**Figure 5:**
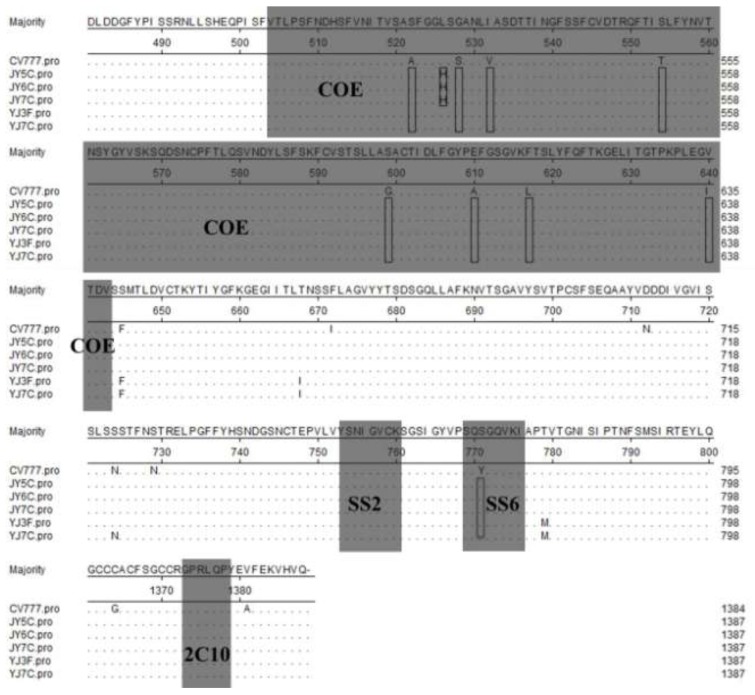
Alignment of amino acid sequences of S proteins of Gansu PEDV strains and reference strains. Ellipses represent the consensus amino acids. Boxes indicate substitution amino acids compared with CV777. Shadows indicate the neutralizing epitopes (COE, SS2, SS6, and 2C10 motif).

The PEDV S protein is a type I glycoprotein. Its neutralizing epitopes contain COE (499–638 aa), SS2 (748–755 aa), SS6 (764–771 aa), and 2C10 (1,368–1,374 aa) [3,4,5], and regions of the alignment sequences (Figure 5) correspond to these regions are COE (504–643 aa), SS2 (753–760 aa), SS6 (769–776 aa), and 2C10 (1,373–1,379 aa). In our study, compared with the vaccine strain CV777, eight mutations (A→S at 517, S→G at 523, V→I at 527, T→S at 549, G→S at 594, A→E at 605, L→F at 612, and I→V at 635) were found in all the five PEDV strains from our study (JY5C, JY6C, JY7C, YJ3F, and YJ7C) and one mutation (L→H at 521) was found in JY5C, JY6C, and JY7C in the neutralizing epitope COE. Compared with the vaccine strain CV777, one mutation (Y→S at 766) was also found in all the five PEDV strains from our study (JY5C, JY6C, JY7C, YJ3F, and YJ7C) in the neutralizing epitope SS6. However, compared with the vaccine strain CV777, no mutations were found in the five PEDV strains from our study (JY5C, JY6C, JY7C, YJ3F, and YJ7C) in the neutralizing epitopes SS2 and 2C10 (Figure 5). Similar to other coronavirus S proteins, the PEDV S protein is a glycoprotein peplomer (surface antigen) on the viral surface, with a pivotal function in stimulating induction of neutralizing antibodies in the natural host. Thus, the S glycoprotein is a primary target for the development of effective vaccines against PEDV. Further research is needed to determine whether the amino acid changes in the Gansu strains from our study result in any antigenicity changes.

### 2.5. Discussion

RT-PCR amplification and sequencing analysis of the full-length PEDV S genes were used to investigate the isolates from diarrhea samples from local pig farms with severe diarrhea in piglets. The variant strains were detected in this study, implying an identical distribution profile for PEDV on pig farms in Gansu, China. The sequence insertions and deletions in the S gene and mutations in the antigenic regions found in variant strains possibly provided stronger pathogenicity and antigenicity to the new PEDV variants that influenced the effectiveness of the CV777-based vaccine, ultimately causing the 2012 outbreak of severe diarrhea in the porcine farms of Gansu Province, China. Future studies should investigate the biological functions of these particular insertions, deletions, and mutations.

## 3. Experimental

### 3.1. Sample Collection

A total of 17 samples (fecal and intestinal) were collected from seven dead piglets showing signs of watery diarrhea and dehydration from two farms in Gansu Province, China (Table 3). These samples were collected individually and placed in separate sterile specimen containers. Samples were homogenized with PBS. The suspensions were vortexed and centrifuged for 10 min at 10,000 × *g*. The supernatants were stored at −80 °C before use. 

**Table 3:** Current farm status in China.

Farm	No. of sows	Vaccination ^a^	Illness rate (%/y)	Mortality rate (%)
(Yongjing Tai Chi Breed Co., Ltd.) YJ	400	Yes	80	60
(Hoggery of Science and Technology Breed Park of Jiugang Hongfeng Company) JY	2000	Yes	80	60

^a^ Sows were vaccinated with divalent inactivated transmissible gastroenteritis (TGE) and porcine epidemic diarrhea (PED) vaccine before delivery.

### 3.2. RNA Extraction and Reverse Transcription

All samples were evaluated by reverse transcription PCR (RT-PCR) using PEDV special primers (Table 4). In brief, viral RNA was extracted from the supernatants of the homogenized samples with TRIzol LS (Invitrogen Co., Carlsbad, CA, USA) according to the manufacturer’s instructions. RT was carried out using random hexamer primers (TaKaRa Bio Inc., Otsu, Japan), and the cDNA was immediately amplified with primers, which were designed based on the sequences of PEDV reference strains (Table 4) under the following conditions: denaturation at 95 °C for 5 min, 35 cycles of denaturation at 94 °C for 30 s, annealing at 52 °C for 30 s, and extension at 72 °C for 90 s. The RT-PCR products were analyzed by 1.5% agarose gel electrophoresis and visualized by ultraviolet illumination after ethidium bromide staining. 

**Table 4:** Amplification primers for the S gene of PEDV in Gansu, China in 2012 ^a^.

Primers	Nucleotide sequence, 5'→3'	Primer location ^b^	Length (bp) ^c^
PEDVS1-P1	CCATTAGTGATGTTGTGTTAG	20, 535–20, 555	1031
PEDVS1-P2	GCACAGCAGCTCCATT	21, 565–21, 550	
PEDVS2-P1	CCACATACCAGAAGGTTTTAG	21, 372–21, 392	1146
PEDVS2-P2	CCAGTAATCAACTCACCCTT	22, 517–22, 498	
PEDVS3-P1	CCCTGAGTTTGGTAGTGG	22, 446–22463	1154
PEDVS3-P2	CATCCGTCTGTAGAGCAAG	23, 599–23, 581	
PEDVS4-P1	CTCATCGGTGGTATGGTGCT	23, 497–23, 516	1355
PEDVS4-P2	AGCAGACTTTGAGACATCTTTGAC	24, 851–24, 828	

^a^ PEDV, porcine epidemic diarrhea virus; P1, forward; P2, reverse. ^b^ Numbers correspond to the nucleotide positions within the CV777 genome. ^c^ Length of PCR products.

### 3.3. Sequence Analysis

Five of 17 pig samples were positive for PEDV by RT-PCR. Sequencing analysis of the full-length S gene was performed for the five samples designated as JY5C, JY6C, JY7C, YJ3F, and YJ7C. In brief, bands of the corresponding size of the gene were excised, and the synthesized DNA was purified using a QIAquick Gel Extraction Kit (QIAGEN, Hilden, Germany) according to the manufacturer’s instructions and sequenced by BGI Company (Peking, China). The five PEDV S gene sequences were aligned with the sequences of 32 previously published PEDV S genes (Table 5) using the DNASTAR, DNAMAN, and MegAlign version 5.0 (DNAStar Inc., Madison, WI, USA) software packages [17]. To investigate their molecular and epidemiological characteristics and to determine their profile of genetic diversity, phylogenetic trees were constructed using molecular evolutionary genetics analysis MegAlign version 5.0 [17] with the neighbor-joining (NJ) method to calculate distance. Bootstrap values were estimated for 1,000 replicates. SignalP 4.1 software [18] was used to predicte the *N*-glycosylated sites.

**Table 5:** Isolates and reference strains used in studies of PEDV outbreak in Gansu, China.

Virus strain	GenBank accession No.	Country and year of isolation
JY5C	KF177254	China 2012
JY6C	KF177255	China 2012
JY7C	KF177256	China 2012
YJ3F	KF177257	China 2012
YJ7C	KF177258	China 2012
CH1	JQ239429	China 2011
CH2	JQ239430	China 2011
CH3	JQ239431	China 2011
CH4	JQ239432	China 2011
CH5	JQ239433	China 2011
CH6	JQ239434	China 2011
CH7	JQ239435	China 2011
CH8	JQ239436	China 2011
CHGD01	JN980698	China 2011
CH-FJND-1-2011	JN543367.1	China 2011
CH-FJND-2-2011	JN315706.1	China 2011
CH-FJND-3-2011	JN381492.1	China 2011
DX	EU031893	China 2007
LZC	EF185992	China 2006
LJB-03	DQ985739	China 2006
JS-2004-2	AY653204	China 2004
CHS	JN547228.1	China 1986
KUN-0901	GU180144	South Korea 2009
KUN-0902	GU180145	South Korea 2009
KUN-0903	GU180146	South Korea 2009
KUN-0904	GU180147	South Korea 2009
KUN-0905	GU180148	South Korea 2009
KUN-0801	GU180142	South Korea 2008
KUN-0802	GU180143	South Korea 2008
Parent DR13	DQ862099	South Korea 2006
Attenuated DR13	DQ462404.2	South Korea 2006
Chinju99	AY167585	South Korea 1999
MK	AB548624.1	Japan 1996
NK	AB548623.1	Japan
KH	AB548622.1	Japan
CV777	AF353511.1	Belgium 1988
Br1-87	Z25483	Great Britain 1993
PUR46-MAD	M94101	USA 1992

## 4. Conclusions

There were few positives (5) by RT-PCR when 17 piglets with water diarrhea and dehydration were sampled. There may be two reasons for this phenomenon. Firstly, inability to amplify virus from all piglets might impact our results. Secondly, the piglets maybe have coinfections that might skew our results. 

Our study of the full-length S gene revealed a more comprehensive distribution profile that reflects the current PEDV status in pig farms in Gansu Province, China, including the presence of strains similar to those from South Korea. These data indicate that the new variant PEDV strains in Gansu Province, which were first found in 2011, may have originated from South China. Thus, certain actions must be taken to prevent the continued transmission of this virus, including the development of novel vaccines for prevention.
